# Investigation of the effect of coffee on body weight, serum glucose, uric acid and lipid profile levels in male albino Wistar rats feeding on high-fructose diet

**DOI:** 10.1186/s42826-019-0024-y

**Published:** 2019-12-18

**Authors:** Teka Obsa Feyisa, Daniel Seifu Melka, Menakath Menon, Wajana Lako Labisso, Mezgebu Legesse Habte

**Affiliations:** 1 Department of Medical Biochemistry, College of Health and Medical Sciences, Haramaya University, Harar, Ethiopia; 2 College of Health Sciences, Addis Ababa University, Addis Ababa, Ethiopia

**Keywords:** Body weight, Coffee, Glucose, High-fructose diet, Lipid profiles, Uric acid

## Abstract

Coffee is one of the most commonly consumed beverages in the worldwide and is assumed to have protective effects against metabolic syndrome. The present study was aimed at investigating the effect of coffee on body weight, serum glucose, uric acid and lipid profile levels in male albino Wistar rats feeding on high fructose diet. A post-test experimental study was conducted on a total of 30 (9–10 weeks old) male albino Wistar rats. The rats were divided into 6 groups: group I (normal control)-fed on standard chow and plain tap water only; group II (fructose control)-fed on standard chow and 20% of fructose solution; group III–VI (treatment groups)-fed on standard chow, 20% of fructose solution and treated with 71, 142, 213 and 284 mg/kg body weight/day of coffee respectively for six weeks. At the end, body weight, serum glucose, uric acid and lipid profile levels were investigated. Data was entered and cleared by epi-data software version 3.1 and analyzed by one way ANOVA followed by Tukey post hoc multiple comparison tests using SPSS V. 23.00. Statistical significance was considered at *p* < 0.05. The results showed that body weight, fasting serum glucose and uric acid levels significantly lowered in rats treated with 213 (*p* = 0.047; 0.049; 0.026) and 284 (*p* = 0.035; 0.029; 0.010) mg/kg body weight/day of coffee compared to fructose control group. Fasting serum triglycide (TG) and low density lipoprotein (LDL-C) levels showed significant reduction in rats treated with 284 mg/kg body weight/day of coffee as compared to fructose control group (*p* = 0.031; 0.046) respectively. In conclusion, treating rats with coffee decreased body weight, fasting serum glucose, uric acid, TC, TG and LDL-C, and increased HDL-C in a dose dependent manner in rats feeding on high fructose diet, suggesting that coffee consumption may be helpful in ameliorating metabolic syndrome.

## Introduction

### Background

Coffee is one of the most frequently consumed beverages in the worldwide and its beneficial effects on human health have become a subject matter of several scientific studies [1]. There are hundreds of variety species of coffee, however, commercially two species are mostly available: *Coffea arabica*, about 70% and *Coffea canephora* [2]. Coffee contains a multitude of chemical substances, many of which are biologically active. Even though the main physiological effects resulting from its consumption are usually ascribed to the presence of caffeine, coffee is also extremely enriched with chlorogenic acids (CGA), melanoidins and diterpenes [3, 4].

Coffee is prepared by several methods, which significantly affect the aroma, flavor and composition of coffee. Many of the coffee compounds, responsible for its unique taste and smell, are formed during the roasting process [5]. On the contrary, the roasting process may cause degradation of several other compounds, including antioxidant polyphenols. Depending on the brewing processes, three major types of coffee can be distinguished: (i) boiled unfiltered coffee, (ii) filtered coffee and (iii) decaffeinated coffee (DC). The property of each type of coffee is related to specific granulation of coffee powder, water/coffee ratio, temperature and brewing time [6].

Many in vitro and in vivo studies, together with epidemiological and human trials have suggested beneficial health effects of coffee. Acute or regular coffee consumption may reduce the risk factors of mortality, cardiovascular disease, T2DM, obesity, liver disease, cancer and many of degenerative diseases such as Alzheimer’s and Parkinson’s [7, 8]. Therefore, the present study was aimed at investigating the effects of coffee on body weight and serum biomarkers of metabolic syndromes such as glucose, uric acid and lipid profile levels in high fructose diet feeding male albino Wistar rats.

## Methods and materials

### Study area

The study was carried out at Biochemistry Department Laboratory, Addis Ababa University, Addis Ababa, Ethiopia.

### Study period and duration

The study was undertaken for a period of 6 months from July 2017 to January 2018.

### Study design

A post-test experimental study was conducted on rat model in order to investigate the effect coffee on body weight, fasting serum glucose, uric acid and lipid profile levels.

### Ethical consideration

The ethical clearance was obtained from Department of Biochemistry Research and Ethical Review Committee (DRERC), by approval letter with Ref. No. of SOM/BCHM/154/2009, meeting No. of DRERC 02/17 and protocol No. of M.Sc. 11/17 issued on March 2/2017. All experimental activities were carried out in accordance with recommendations from the declaration of nationally and internationally conventional standards for the employment of experimental animals, and code of ethics of animal experiments, which comply with scientific and ethical guidelines.

### Study variables

Independent variable was coffee administration. Dependent variables were body weight, Serum glucose, uric acid and lipid profile levels.

### Sample size determination

Sample size determination was based on WHO standard, which recommends that each treatment group of experimental animal should be at least 5 animals [9]. The study was conducted on 6 groups of rats. Therefore, the total sample size was 6 groups × 5 rats = 30 rats.

### Inclusion and exclusion criteria

Inclusion criteria were age (9-10 weeks old) and normal body weight (< 250 g).

Exclusion criteria were suffering from diarrhea, or any observable physical abnormality before treatment.

### Experimental animals and protocol

A total of 30 male albino Wistar rats of 6–7 weeks were obtained from Addis Ababa University, department of Pharmacology. The experimental rats were placed in a plastic cage with stainless steel cover (5 rats/cage) and housed in biochemistry animal laboratory with optimum temperature (24 + 1 °C), relative humidity, optimum ventilation and 12-h light-dark cycle. They were standardized for their feeding behavior and observed continuously for three weeks. Until the initiation of the experiment, all rats were provided with free access to standard chow and plain tap water. When the rats became 9–10 weeks old (adult), they were randomly assigned into six groups. Each rat in the given group was identified by giving a number on its tail by permanent marker. Prior to the initiation of experiment, their body weight was measured by triple beam balance and ranged from 205 g to 245 g, with mean value of 225.4 ± 11.5 g. Then the rats were treated as follows:

- **Group I (Normal control group):** fed on standard chow and plain tap water only.
- **Group II (Fructose control group):** fed on standard chow and 20% (w/v) of fructose solution.
- **Group III (Treatment group):** fed on standard chow and 20% (w/v) of fructose solution, and treated with 71 mg/kg BW/day of coffee.
- **Group IV (Treatment group):** fed on standard chow and 20% (w/v) of fructose solution, and treated with 142 mg/kg BW/day of coffee.
- **Group V (Treatment group):** fed on standard chow and 20% (w/v) of fructose solution, and treated with 213 mg/kg BW/day of coffee.
- **Group VI (Treatment group):** fed on standard chow and 20% (w/v) of fructose solution, and treated with 284 mg/kg BW/day of coffee.

### Preparation of fructose solution and coffee

#### Preparation of fructose solution

Fructose used in this experiment was pure crystalline, SIGMA-ALDRICH, USA, purchased from India. Twenty percent (20%, W/V) of fructose solution [10] was prepared on daily basis and substituted for tap water in five groups of the rats (group II to group VI).The net consumption of each group was recorded in mL/day.

#### Preparation of coffee and dosage calculation

Coffee used as a treatment in the present study was *Coffea arabica* (TO.MO.CA Coffee packet), purchased from coffee shop in Addis Ababa. TO.MO.CA stands for Torrefazione Moderna Cafe (Italian), translated directly as modern coffee roasting. Taking into account the coffee brewing process in Ethiopia, boiled, unfiltered coffee was prepared following the instruction written on the packet (by adding 10 g of coffee powder into 180 mL of hot water. To estimate the amount of dissolved coffee powder, indirect method of measurement was used (the residue of the coffee powder was measured and subtracted from the total added powder). Accordingly, the volume of the coffee solution after boiling and decantation was 120 mL. The amount of undissolved air-dried residue of coffee powder was 5.2 g, so the dissolved amount was calculated as 10 g–5.2 g =4.8 g. The volume of 1 cup of coffee was as recently used by Lelyana and Lelyana et al. (0.36 mL/200 g body weight/day) in rats, which is equivalent 125 mL/70 kg body weight/day of coffee in humans [9, 11]. In our study, the average body weight (BW) of the experimental rats was 225 g.

Accordingly, 16 mg/225 g BW/day of coffee was considered as a single dose of coffee. Similarly, 0.8, 1.2 and 1.6 mL/225 g BW/day (32 mg, 48 mg and 64 mg/225 g BW/day) were considered as a double, triple and quadruple doses of coffee. For the seek of convenience, these doses were converted to standard unit (mg/kg) as 71, 142, 213 and 284 mg/kg BW/day respectively.

The total volume of coffee administered to treatment groups and clear warm water to normal and fructose control group by oral gavage was 2 mL. The oral gavage was performed by the principal investigator between 09:00 and 10:00 a.m. every day. The experiment was conducted for a period of 6 weeks. No rat died as a result of the treatment or other causes throughout the experiment.

### Data collection

#### Measurement of body weight

Body weight of the experimental rats was measured by triple beam balance capable of measuring 610 ± 0.1 g at initial and weekly during the experiment and recorded to the corresponding code of each rat in the group. However, due to the fluctuation of body weight between weeks, only the initial and final body weights were considered for final statistical test.

#### Blood sample collection

At the end of the sixth week, the rats were fasted overnight by removing fructose solution and and standard chow, however, tap water was supplied for all groups of the rats. After overnight fasting the rats were anesthetized with diethyl ether. Blood sample was collected by cardiac puncture and the rats were killed by exsanguination. To prepare serum, the blood sample was transferred into serum separator tube (SST) and left to clot at room temperature for 30 min immediately following collection. Subsequently, the clotted blood sample was centrifuged at 2000 rpm for 15 min. Finally, the serum was transferred into necked tube and stored at − 80 °C [12] until the analyses were performed.

#### Laboratory tests

Serum glucose, uric acid, TC, HDL-C and TG were determined by an enzymatic colorimetric methods using fully automated analyzer (Mindary BS-200E); LDL-C was calculated using Frieldwald’s formula.

#### Data entry and analysis

All data were entered and cleared by epi-data software version 3.1 and exported to SPSS (statistical Package for Social Science) software version 23.0 for statistical analysis. Normality distributions were assessed by Shapiro-Wilks test and plots (stem-and-leaf and histogram).One-way analysis of variance (ANOVA) was done to determine statistical differences among all groups of the study. Pairwise comparisons were conducted by Tukey post hoc multiple comparison tests.The results of the data were presented as mean ± standard deviation (SD). The *p*-values < 0.05 were considered statistically significant.

## Results

### Effects of coffee on energy intake

**Fig. 1:**
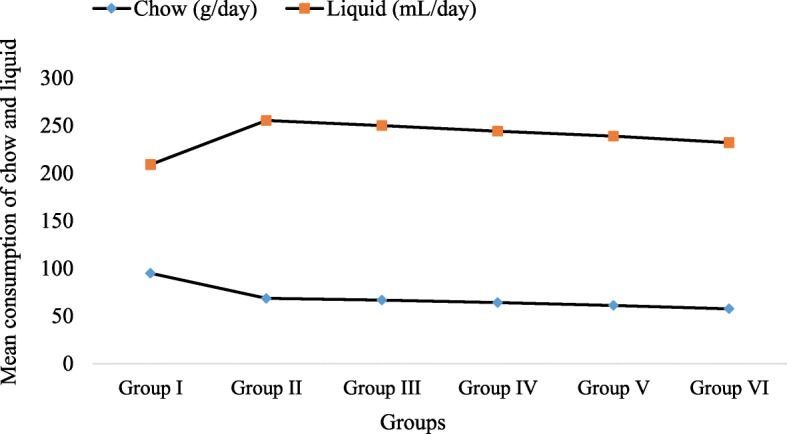
The mean consumption of chow and liquid of the rats. The values are expressed as mean. Sample size (n) is 5 for each group. Liquid - refers to fructose solution for all groups, except for group I where it refers to plain tap water. Group I – Normal control group; Group II-Fructose control group; Group (III- VI) –Treatment groups (received 71, 142, 213 and 284 mg/kg BW/day of coffee) respectively

The rats treated with 284 mg/kg BW/day of coffee consumed lower amount of chow compared to fructose control group (57.5 g/day vs 68.5 g/day) and normal control (57.5 g/day vs 95.5 g/day).The results also shows that the mean liquid intake in rats treated with 284 mg/kg BW/day of coffee was lower when compared to fructose control group (174.5 mL/day vs 187 mL/day). Since the chow and fructose solution were supplied by treatment group, no statistical test was done (Fig. 1).

### Effect of coffee on body weight

**Fig. 2:**
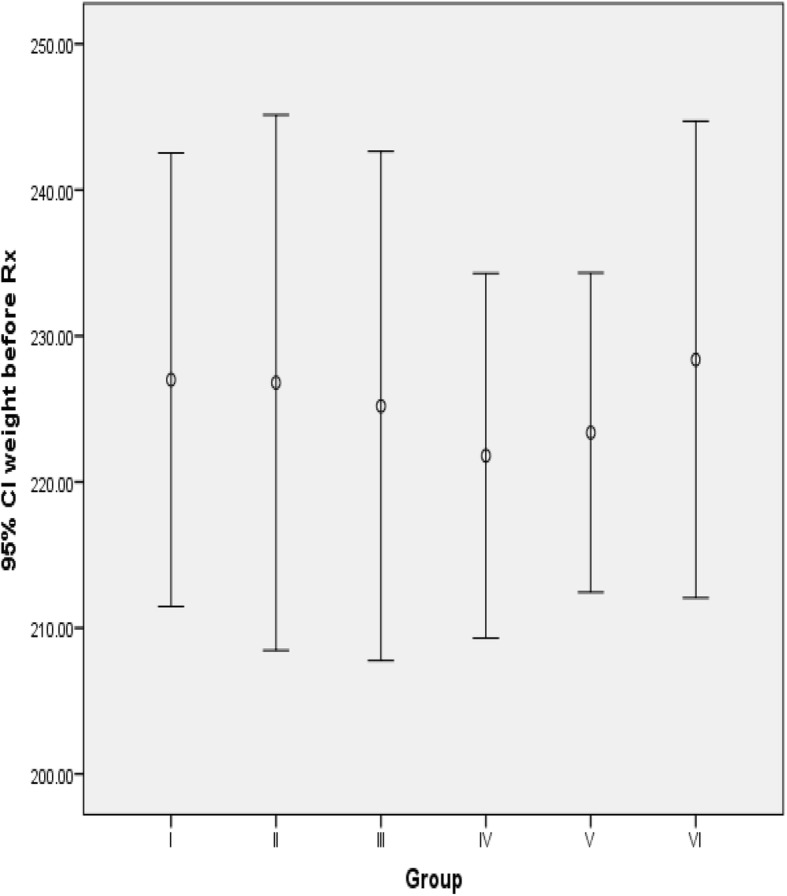
Initial body weight of the rats. Values are expressed as mean ± SD. Sample size (n) is 5 for each group I- Normal control group; II-Fructose control group; III-VI -Treatment groups (received 71, 142, 213 and 284 mg/kg BW/day of coffee) respectively

Initially, the body weight of the rats was statistically similar among all groups (*p* = 0.96) (Fig. 2).

**Fig. 3:**
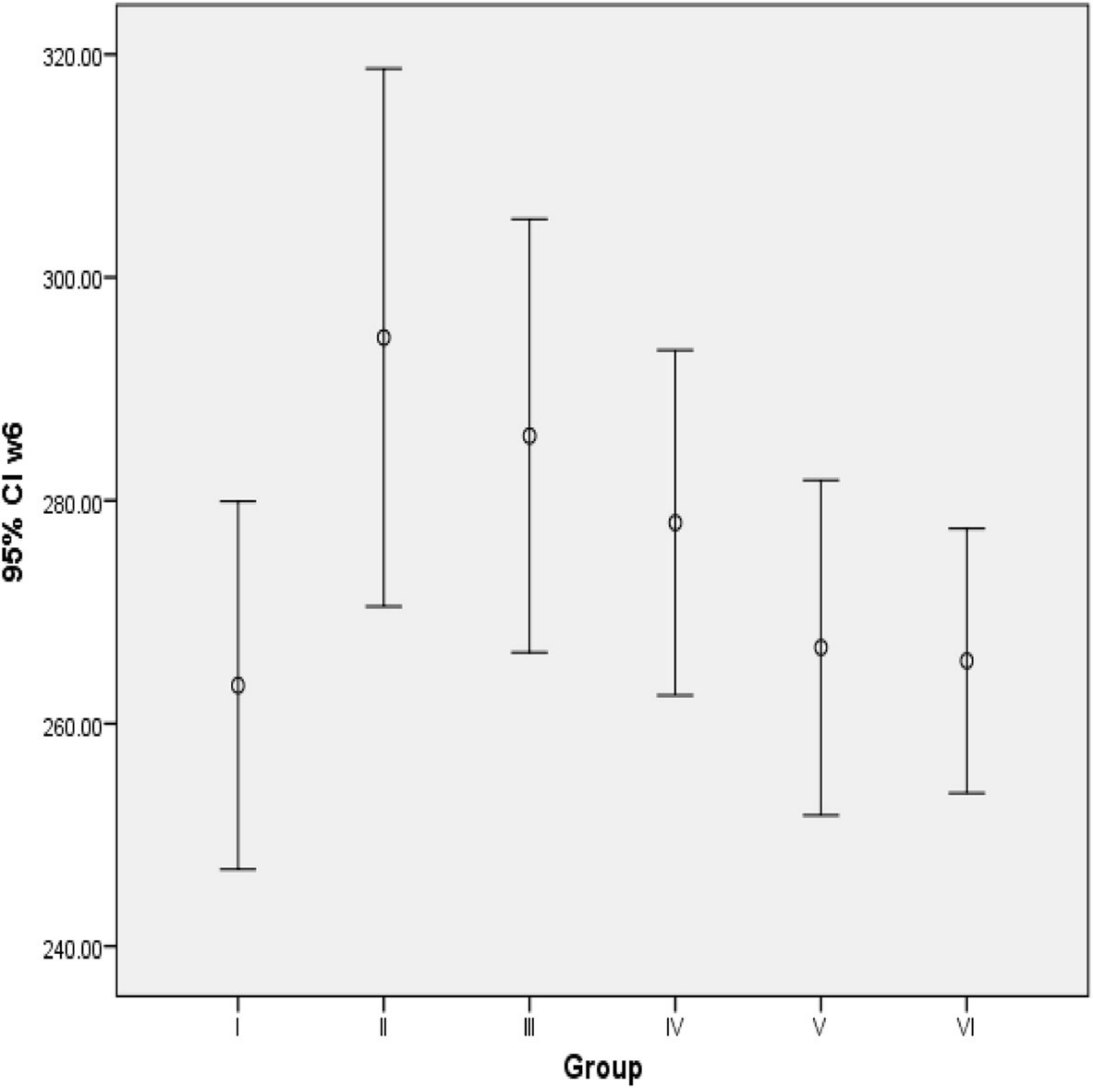
Final body weight of the rats. Values are expressed as mean ± SD. Sample size (n) is 5 for each group I- Normal control group; II-Fructose control group; III-VI -Treatment groups (received 71, 142, 213 and 284 mg/kg BW/day of coffee) respectively

At end of the sixth week, the body weights of rats treated with 213 and 284 mg/kg BW/day of coffee significantly reduced compared to fructose control group (*p* = 0.047; 0.035) respectively. In addition, body weight of fructose control group was significantly higher compared to normal control group (*p* = 0.020) (Fig. 3).

### Effect of coffee on serum glucose and uric acid levels

Treating the rats with 213 and 284 mg/kg BW/day of coffee significantly decreased fasting serum glucose (*p* = 0.049; 0.029) and uric acid (*p* = 0.026; 0.010) levels compared to fructose control group. Fasting serum glucose and uric acid levels were significantly increased in fructose control group when compared to normal control (*p* = 0.007, 0.002) respectively (Table 1).

**Table 1:** Fasting serum glucose and uric acid levels of the rats

Variable (mg/dL)	Groups						F	*P*
	I	II	III	IV	V	VI		
Glucose	74.2 ± 8	94.4 ± 9.6^abc^	88.8 ± 9	81 ± 5.9	78.4 ± 8.4	77.2 ± 7.5	4.5	0.005*
Uric acid	1.32 ± 0.3	2.18 ± 0.3^abc^	1.7 ± 0.4	1.64 ± 0.3	1.52 ± 0.26	1.44 ± 0.3	4.7	0.004*

_*_-Indicates significant differences among all groups at *p* < 0.05 as tested by one-way ANOVA. Superscript letters (a, b and c) indicate significant differences compared to group I, V and VI respectively at *p* < 0.05 as tested by Tukey post hoc multiple comparisons

### Effect of coffee on serum lipid profile levels

Fasting serum TG and LDL-C levels were significantly lower in rats treated with 284 mg/kg BW/day of coffee compared to fructose control group (*p* = 0.031; 0.046) respectively. Similarly, significant elevation of fasting serum TG (*p* = 0.013) and LDL-C (*p =* 0.007) levels were also found in fructose control group when compared to normal control group. Significantly increased fasting serum LDL-C level was also observed in rats treated with 71 mg/kg BW/day of coffee as compared to normal control group (*p* = 0.046), (Table 2).

**Table 2:** Fasting serum lipid profile levels of the experimental rats

Variable (mg/dL)	Groups						F *P*	
	I	II	III	IV	V	VI		
TC	101.4 ± 13.2	120.2 ± 12.4	118.6 ± 8.8	113.2 ± 10.2	106.6 ± 9.4	103.8 ± 10.7	2.6	0.051
HDL-C	44 ± 5.5	33.4 ± 5.5	37.6 ± 4.5	39 ± 4.3	39.8 ± 6.8	41 ± 7.3	2	0.132
TG	116.8 ± 11	147.8 ± 17.8^ab^	142.6 ± 13.2	135 ± 15.4	125.4 ± 10.6	120 ± 9.8	4.5	0.005*
LDL-C	34 ± 9.5	57.2 ± 10.4^ab^	52.5 ± 4.4^a^	47.2 ± 13.5	41.7 ± 6.7	38.8 ± 8.8	4.4	0.006*

HDL-C- High density lipoprotein cholesterol; LDL-C- Low density lipoprotein cholesterol; TC- Total cholesterol; TG- Triglycerides. Sample size (n) is 5 for each group._*_ **-**Indicates significant difference among all groups at *p* < 0.05 as tested by one-way ANOVA. Superscript letters (a) - indicates significant differences compared to group I; (b) - indicates significant differences compared to group VI at *p* < 0.05 as tested by Tukey post hoc multiple comparisons

## Discussion

The obtained results indicated that significantly decreased body weight gain was recorded in rats treated with 213 and 284 mg/kg BW/day of coffee at the end of sixth week as compared to fructose control group (*p* = 0.047; 0.035) respectively. These results suggest that the effect of coffee on body weight is dose dependent. In line with this finding, Ismail et al. reported that male albino rats fed on different preparations of Turkish, Arabian and instant coffee had significantly smaller weight gain than normal control [13]. Similar finding was also reported by Mohmoud et al. in which feeding on different doses of Arabic coffee for 30 days lowered body weight in rats fed on basal diet [14]. The possible mechanism by which coffee prevented higher body weight gain in the present study could be by increasing lipolysis via catecholamines [15, 16]. Caffeine might also have caused body weight loss by increasing physical activity.

In addition, coffee had decreased energy intake, which might in turn had led to decreased in the body weight gain. In comparison with normal control group, fructose control group had statistically significant body weight gain (*p* = 0.020). This result is in line with Tanaka et al.*’s* report in which feeding on 20% fructose solution in tap water for 8 weeks had brought statistically significant body weight gain in both sexes of rats [1]. As demonstrated in both short-term and long-term studies, fructose consumption results in decreased circulating levels of insulin and leptin and increased caloric intake [17].

Treating rats with 213 and 284 mg/kg BW/day of coffee had significantly reduced fasting serum glucose levels as compared to fructose control group (*p* = 0.049; 0.029) respectively. Consistent with these findings, Li et al. reported that CGA significantly lowered fasting glucose levels in golden hamsters [18]. In addition, Takami et al. reported moderate coffee consumption in human subjects was associated with a significantly lower odds ratio for high plasma glucose [19].The overall effects of coffee on glucose level in the present study revealed good implication to support the hypothesis that states, “Heavy coffee consumption has been associated with a lower risk of diabetes,” [20].

Most probably, coffee prevented increase in glucose level due to its main component, caffeine, which inhibits adenosine receptors that stimulates hepatic glucose production through the activation of A2B adenosine receptors [21]. Caffeine might also have stimulated glucose transport through activation of cyclic AMP-dependent protein kinase α-1 [20]. Another mechanism could be via selective inhibition of hepatic glucose-6-phosphatase, a rate-limiting enzyme of gluconeogenesis, by CGA of coffee [3, 22].

Fasting serum glucose was significantly higher in fructose control group than in normal control group (*p* = 0.007). This finding is in agreement with Sandeva et al.*’s* study, which reported that serum glucose level was significantly increased in male and female rats fed on 20% of fructose solution in drinking water for 8 weeks [23].

It was found that fasting serum uric acid levels were significantly lower in rats treated with 213 and 284 mg/kg BW/day of coffee compared to fructose control group (*p* = 0.026; 0.010) respectively. Similar studies by Lelyana and Lelyanaet al. reported that coffee had non-significantly reduced serum uric acid in obese rats and mice with hyperuricemia respectively [9, 11]. In addition, Mahmoud et al. reported that different doses of Arabic coffee had lowered serum uric acid levels in experimental rats fed on basal diet [14]. In comparison with mentioned findings, the significant decrease in fasting serum uric acid found in the present study might be due to variation in the doses of coffee, study design and/or duration. Coffee consumption may lower serum uric acid levels and risk of gout via various mechanisms. The main mechanism is via competitive inhibition of xanthine oxidase by caffeine (1,3,7-trimethyl-xanthine), [24]. The other mechanism is probably by a diuretic action of caffeine that might affect serum uric acid concentration through increased excretion in urine [21].

Evaluation of fasting serum lipid profiles showed that treating rats with 284 mg/kg BW/day of coffee had significantly lowered fasting serum levels of TG and LDL-C when compared to fructose control group (*p* = 0.031; 0.046) respectively. Consistent with these findings, Gomes et al. reported that feeding of 7.2 mL/kg of body weight for 41 days significantly lowered lipid percentage in hyperlipidemic diet feeding rats [25]. In addition, Li et al. reported CGA significantly lowered the levels of fasting serum TG, FFA, TC, and LDL-C in golden hamsters [18].

Fasting serum levels of TC and HDL-C were non-significantly lower and higher respectively, in rats treated with 213and 284 mg/kg BW/day of coffee compared to fructose control group (Table 3).These results are in agreement with Karabudak *et al*.’s finding that reported Turkish and instant coffee consumption did not significantly affect serum levels of TC among Turkish subjects [26]. On the other hand, in Mohmoud et al.’s study,Arabic coffee had significantly decreased serum TC levels in experimental rats fed on basal diet [14]. In contradiction to our findings, few studies reported significant elevation of serum TL, TC, TG and LDL-C, while a significant decrease of HDL-C in rats fed diet supplemented with low or high dose of coffee.

The causes for these variations are unclear. However, it may be due to the variations in the mode of coffee administration. Lipid lowering effects of coffee found in the present study could be related to caffeine, (methylxanthine) which stimulates lipolysis by increasing cellular levels of cAMP through antagonism adenosine receptors (A1) and inhibition of phosphodiesterase activity [25, 27].

Feeding rats on 20% of fructose solution for six weeks significantly increased fasting serum levels of TG in fructose control (*p* = 0.013); LDL-C in fructose control (*p* = 0.007) and 71 mg/kg BW/day of coffee treated(*p* = 0.046) groups compared to normal control group.The fasting serum levels of TC (*p =* 0.106) and HDL-C (*p* = 0.073) were non-significantly higher and lower,respectively in fructose control group than in normal control group (Table 3).Fasting serum levels of TC, TG, and LDL-C were possibly increased in fructose control group as metabolism of fructose bypasses the enzyme phosphofructokinase and unregulated the flow of fructose-derived substrates (glycerol-3- phosphate and acetyl-CoA) into lipogenesis [28].

## Conclusions

Treating rats with Arabic coffee for six weeks was found to reduce body weight, fasting serum glucose, uric acid and lipid profile (TC, TG and LDL-C) levels in a dose dependent manner in male albino Wistar rats feeding on 20% of fructose solution. Notably, 213 and 284 mg/kg BW/day of coffee significantly reduced body weight, fasting serum glucose and uric acid levels. In addition, 284 mg/kg BW/day of coffee significantly decreased fasting serum TG and LDL-C levels. Coffee treatment also lowered the fasting serum levels of TC and increased HDL-C although not significant. Overall, the cumulative findings of the present study suggest that coffee consumption may be helpful in ameliorating metabolic syndromes and its associated complications such as obesity, diabetes, inflammation and cardiovascular diseases.

## Data Availability

All necessary data and materials related to the article are included in the manuscript.

## Abbreviations

ANOVA: Analysis of Variance
BW: Body Weight
cAMP: Cyclic Adenine Monophosphate
CGA: Chlorogenic Acid
FFA: Free Fatty Acid
gm: Gram
HDL-C: High Density Lipoprotein Cholesterol
Kg: Kilogram
LDL-C: Low Density Lipoprotein Cholesterol
mg/dL: Milligram per deciliter
rpm: Revolution per Minute
SD: Standard Deviation
T2DM: Type 2 Diabetes Mellitus
TC: Total Cholesterol
TG: Triglycerides
TL: Total Lipid
UA: Uric Acid
W/V: Weight Per Volume
